# Selection and Evaluation of Reference Genes for Reverse Transcription-Quantitative PCR Expression Studies in a Thermophilic Bacterium Grown under Different Culture Conditions

**DOI:** 10.1371/journal.pone.0131015

**Published:** 2015-06-26

**Authors:** Kathleen D. Cusick, Lisa A. Fitzgerald, Allison L. Cockrell, Justin C. Biffinger

**Affiliations:** 1 National Research Council Associateship, US Naval Research Laboratory, 4555 Overlook Ave., SW, Washington DC, 20375, United States of America; 2 Chemistry Division, US Naval Research Laboratory, 4555 Overlook Ave., SW, Washington DC, 20375, United States of America; Northwestern University, UNITED STATES

## Abstract

The phylum Deinococcus-Thermus is a deeply-branching lineage of bacteria widely recognized as one of the most extremophilic. Members of the *Thermus* genus are of major interest due to both their bioremediation and biotechnology potentials. However, the molecular mechanisms associated with these key metabolic pathways remain unknown. Reverse-transcription quantitative PCR (RT-qPCR) is a high-throughput means of studying the expression of a large suite of genes over time and under different conditions. The selection of a stably-expressed reference gene is critical when using relative quantification methods, as target gene expression is normalized to expression of the reference gene. However, little information exists as to reference gene selection in extremophiles. This study evaluated 11 candidate reference genes for use with the thermophile *Thermus scotoductus* when grown under different culture conditions. Based on the combined stability values from BestKeeper and NormFinder software packages, the following are the most appropriate reference genes when comparing: (1) aerobic and anaerobic growth: TSC_c19900, *polA2*, *gyrA*, *gyrB*; (2) anaerobic growth with varied electron acceptors: TSC_c19900, *infA*, *pfk*, *gyrA*, *gyrB*; (3) aerobic growth with different heating methods: *gyrA*, *gap*, *gyrB*; (4) all conditions mentioned above: *gap*, *gyrA*, *gyrB*. The commonly-employed *rpoC* does not serve as a reliable reference gene in thermophiles, due to its expression instability across all culture conditions tested here. As extremophiles exhibit a tendency for polyploidy, absolute quantification was employed to determine the ratio of transcript to gene copy number in a subset of the genes. A strong negative correlation was found to exist between ratio and threshold cycle (C_T_) values, demonstrating that C_T_ changes reflect transcript copy number, and not gene copy number, fluctuations. Even with the potential for polyploidy in extremophiles, the results obtained via absolute quantification indicate that relative quantification is appropriate for RT-qPCR studies with this thermophile.

## Introduction

Organisms that thrive in environments classified as “extreme” from an anthropogenic viewpoint are commonly referred to as “extremophiles.” The biomolecules (proteins, nucleic acids, membranes, and small molecules) and molecular mechanisms of these organisms have evolved to successfully function under conditions typically viewed as abiological, such as extreme temperatures, pH, salinities, and pressures [1]. The enzymes from thermophilic microbes are of particular interest due to their increased activity and stability in comparison to mesophilic or synthetic enzymes currently employed for the production of food, detergents, drugs, and paper [2]. The phylum Deinococcus-Thermus is a deeply-branching lineage of bacteria widely recognized as one of the most extremophilic. Representatives from both the *Deinococcus* and *Thermales* orders are of great interest due to the ability of their proteins and associated cellular components to function under extreme conditions such as highly elevated temperatures and ultraviolet radiation exposure. For example, the well-known and now commonly-employed thermostable enzyme *Taq* DNA polymerase was isolated from a *Thermus aquaticus* strain from a hot spring in Yellowstone National Park [3].

Members of the *Thermus* genus in general are of major interest due to both their bioremediation and biotechnology potentials [4]. *Thermus* sp. are able to reduce heavy metals and transition to anaerobic respiration under conditions of oxygen deprivation, and are able to produce industrially important thermostable enzymes while displaying resistance to denaturing physical and chemical factors [5]. *T*. *scotoductus* in particular has been found to reduce a variety of metals such as Fe(III), Cr(VI), Mn(IV), U(VI), and Co(III) [6–9], the benefits of which include the bioremediation of heavy metals; the removal of organic contaminants and mobilization of phosphates from water supplies; and the reduction of global warming due to the diversion of electrons away from methane producers under anaerobic conditions [4].

Gene expression studies provide the potential to identify novel molecular mechanisms and pathways in extremophiles, such as the ability to evolve and thrive in these environments, as well as the identification of novel enzymes for use in biotechnological and bioremediation strategies. Our lab is currently utilizing *T*. *scotoductus* grown (1) anaerobically to examine the correlations between metal respiration and nanoparticle generation and (2) aerobically to determine the physiological and biochemical changes associated with dielectric heating (Cockrell *et al* In Prep). Other studies have utilized these species grown aerobically when studying various proteins [10] or physiological parameters associated with heavy metal reduction [8, 9] as well. However, little to no molecular information exists as to the pathways and mechanisms utilized by *T*. *scotoductus* during metal reduction and aerobic versus anaerobic growth in general. A sequenced genome [11] provides valuable tools to gain insights as to genes and pathways used under these conditions.

Reverse-transcription quantitative PCR (RT-qPCR) has become a common tool for the measurement of gene expression due to its specificity, sensitivity (>10^7^-fold dynamic range), and amenability for high throughput sample processing due to the 96- and 384-well plate configuration of most qPCR instruments [12]. Two types of quantification are used to measure changes in gene expression levels: absolute and relative. Absolute quantification determines the precise amount of target, typically expressed as copy number, while relative quantification measures the ratio between target and reference gene transcripts. In absolute quantification, the amount of target is calculated using an external standard curve of known concentration. Using relative quantification, target gene expression levels are normalized to endogenous reference or housekeeping genes [13]. The selection of an appropriate reference gene is a key parameter when relative quantification is employed [14]. Ideally, the expression of this gene remains stable under the experimental conditions [15]. However, it is becoming increasingly evident that commonly employed reference genes—thought to be constitutively expressed and minimally regulated—in fact display considerable variance across time, cell type, or experimental treatment [16]. This may be attributed in part to the fact that some housekeeping proteins are involved in functions other than basal cell metabolism [17, 18] or that housekeeping metabolism is a very dynamic process that is efficient at adapting to different growth conditions [19]. Additionally, the use of multiple reference genes is now widely advocated, as the use of a single reference gene has been shown to result in relatively large errors [15].

Data indicate that typical bacterial reference genes–for example, the *rpo* subunits and 16S rRNA gene–may not be the most appropriate [20]. Various studies dedicated to reference gene selection have been performed with mesophilic bacterial species in recent years. These include (with the most appropriate genes listed in parentheses): the human gut-associated *Bifidobacterium adoloescentis* exposed to bile (*gyrA* and *sdhA*) [20]; *P*. *aeruginosa* from cystic fibrosis patients (genes coding for the conserved hypothetical proteins PA2875 and PA3340) [21]; *Clostridium ljungdahlii* (*gyrA*, *rho*, and *fotl*) across multiple culture conditions [22]; the fish pathogen *Francisella noatunensis* (outer membrane protein *fopA*, cell division protein *ftsZ*, and DNA polymerase *polA*) [23]; the nitrogen-fixing endophyte *Gluconacetobacter diazotrophicus* grown under different carbon sources (*rho*, 23S rRNA, and *rpoD*) [24]; the group B streptococci species *Streptococcus agalactiae* (*recA*) [25]; *Lactobacillus casei*, a potential probiotic strain (GAPD, *gyrB*) [26]; and the marine bacterium *Zobellia galactanivorans* (serine/glycine hydroxymethyltransferase A *glyA*, NADP-dependent isocitrate dehydrogenase *icdA*, guanylate kinase *gmkA*) [27].

Limited studies exist that focus on reference gene selection in extremophiles, with only a single dedicated study performed in recent years for the acidophile *Acidithiobacillus ferrooxidans* [28]. Due to their unique physiologies, suitable reference genes in extremophiles may differ from those identified in mesophiles. For example, temperature stress in multiple mesophilic species identified elongation factor TUF 6-phosphogluconate dehydrogenase (6PGDH) as most stably expressed [29]–yet the temperature to which the species were exposed (40–42.5°C) is below the limit at which thermophiles thrive. Thus, the selection and evaluation of appropriate reference genes is a prerequisite when utilizing expression profiling to identify key pathways and mechanisms in *Thermus* sp. grown under different environmental conditions.

One additional issue that must be addressed for gene expression profiling under different growth conditions or over time in both extremophiles and mesophiles is that of genome copy number. In general, bacteria are thought of as monoploid (i.e. harboring only a single copy of their chromosome). However, the number of polyploidy “exceptions” may now exceed the monoploidy “rule.” [30]. A survey of 11 species of proteobacteria demonstrated that only three of the species were monoploid, with the gamma-proteobacterium *Pseudomonas putida* containing 20 copies of its genome [31]. The extremely thermophilic bacterium *Thermus thermophiles* HB8 has been shown to display polyploidy, harboring multiple genomic copies in a cell [32]. Similarly, *Deinococcus radiodurans*, an exceptionally radioresistant bacterium closely related to *Thermus* sp., has also been shown to be a polyploidy organism, possessing four genome copies per cell in the stationary phase and up to 10 copies per cell during exponential growth [33, 34]. Even species typically classified as monoploid may display polyploidy, as genome copy number may vary depending on growth rate: cells that are growing more quickly have multiple initiation events, and therefore multiple genomes per cell. This has been shown to be the case with *P*. *aeruginosa*, which contains 2–3 copies of the genome per cell during exponential growth, and 1–2 copies during stationary phase [35]. Genome copy number in the model bacterium *Escherichia coli*, long thought to be monoploid, has also been shown to increase to 6–7 copies as a function of growth rate, while the cyanobacterium *Synechocystis* possesses 218 genome copies in the exponential phase and 58 during stationary phase [30].

It is necessary to determine whether changes in gene expression are an accurate portrayal of transcript fluctuations, or whether changes in transcript abundance are derived from gene copy number fluctuation due to potential polyploidy. This can be accomplished via absolute quantification of both transcript and gene copy numbers standardized to a unit value such as cell number, volume of sample, or mass. This transcript-to-gene copy number ratio provides a means to determine whether transcript variation is derived from changes in gene or transcript copy number. We previously used this approach to identify new reference genes and confirm that relative quantification was an appropriate method for assessing gene stability with fluctuations in gene copy number with the fungus *Neurospora crassa*, which possesses multinucleated cells at various stages during growth [36]. Little to no information exists as to the appropriate reference genes to use in *Thermus* sp. gene expression studies. Additionally, the issue of gene expression changes as a result of fluxing genome copy number in extremophiles has not been addressed. The objective of the current study was to examine the potential of a suite of genes for use as reference genes over time and/or under different culture conditions in *T*. *scotoductus*. The expression stabilities and appropriate rank of these 11 genes were evaluated using the BestKeeper [37] and NormFinder [38] software tools. Transcript and gene copy numbers were then examined using absolute quantification in a subset of these genes in order to determine whether instances of expression instability were a result of changes in transcript or gene copy number.

## Materials and Methods

### Organism Maintenance

*Thermus* sp. SA-01 (ATCC 700910) was obtained from the American Type Culture Collection. Lyophilized stocks were initially reconstituted in Castenholtz TYE (ATCC medium 416) per ATCC recommendations. Stock cultures were stored at -80°C in 20% glycerol. Cultures were routinely cultured under aerobic conditions at 65°C by inoculation of a single colony into TYG broth (5 g tryptone, 3 g yeast extract, 1 g glucose per liter per the methods of [7]) followed by 1% inoculation into the desired culture medium. For anaerobic culture experiments, the aerobic TYG culture was passed through one sub-culture consisting of a defined basal medium [6] amended with 30 mM glucose as the carbon source and 10 mM sodium nitrate as the terminal electron acceptor.

### Culture Conditions and Cell Harvesting

*Thermus* sp. was incubated under a range of culture conditions in order to assess growth and test the expression stability of a suite of potential reference genes over time or when in the presence of different electron donors and acceptors. All experiments were conducted at 65°C. Table 1 summarizes the culture conditions. A minimum of three of biological replicates were performed under each condition. Briefly, reference gene expression stability was assessed (1) over time in a specific culture condition or (2) at specific time point(s) between different growth conditions. Anaerobic growth experiments were carried out in 50 mL Hungate tubes (Bellco Glass, Inc.) filled with 10 mL of the appropriate medium (described below). Filter-sterilized carbon sources and terminal electron acceptors were added to the appropriate concentrations, and the tubes sealed with black butyl rubber stoppers under an N_2_/CO_2_ atmosphere. Aerobic growth experiments were carried out in 50 mL glass tubes filled with 10 mL of TYG. Both anaerobic and aerobic cultures were grown under mild agitation (70 rpm). Growth was also compared between two different heating methods: incubation in an oven (i.e. traditional incubator) versus heating within a synthetic microwave (CEM) set to a power of 300W. These experiments utilized 60 mL TYG in 100 mL Teflon digestion vessels (CEM) with no agitation. At each time point, cells were collected for DNA and RNA extraction. For RNA extraction, 4 mL of sample was centrifuged for 3 min at 12,000 X g, the pellet washed in 500 μL RNAProtect Bacteria Reagent (Qiagen), centrifuged for an additional 3 min at 12,000 X g, and re-suspended in a final volume of 40 μL RNAProtect Bacterial Reagent. Samples were stored at -80°C until total RNA extraction.

**Table 1:** Experimental growth conditions and time points for gene expression profiling in *T*. *scotoductus*.

Condition	Medium	Electron Donor, Acceptor	Sampling Points
Anaerobic	Basal (10 mL)	30 mM glucose,10 mM nitrate	multiple time points over 24 h growth
Anaerobic	Basal (10 mL)	30 mM lactate,10 mM nitrate	multiple time points over 24 h growth
Anaerobic	Basal (10 mL)	30 mM glucose,10 mM Fe(III) citrate or 10 mM nitrate	single time point at 24 h
Aerobic	TYG (10 mL)	N/A	multiple time points over 24 h growth
Aerobic—Oven	TYG (60 mL)	N/A	multiple time points over 48 h growth
Aerobic—Microwave	TYG (60 mL)	N/A	multiple time points over 48 h growth

### DNA Extraction

Genomic DNA was extracted from biological triplicates using the DNeasy Blood and Tissue Kit (Qiagen, Valencia, CA) following the manufacturer’s instructions for pre-treatment of Gram-negative bacteria. The initial Proteinase K incubation was performed for 2 hours at 56°C, with frequent vortexing. Samples were eluted in a final volume of 100 μL Buffer AE and stored at -20°C until use. DNA concentration and purity were assessed using the Nanodrop-2000 (Thermo Fisher Scientific, Wilmington, DE). DNA was diluted 1:5 in nuclease-free water prior to use.

### RNA Isolation and cDNA synthesis

An optimized protocol was developed for total RNA extraction from *Thermus* sp. based on the general methodology of the RNeasy Mini Kit (Qiagen, Valencia, CA). Briefly, 180 μL of lysozyme (15 mg/mL) re-suspended in TE Buffer (30 mM Tris-HCl, 1 mM EDTA, pH 8) plus 20 μL Proteinase K (Qiagen) was added to each cell pellet (previously re-suspended in 40 μL RNAProtect) and samples incubated on a shaking platform (125 rpm) for 25 min at room temperature. Seven hundred μL Buffer RLT containing β-mercaptoethanol was then added to each sample. The remaining steps followed the manufacturer’s protocol. The optional on-column DNase digestion was extended to 1 h. An additional spin at top speed (21,130 X g) for 2 min was included immediately prior to elution in 40 μL RNase-free water. Total RNA concentration and purity was measured using the Nanodrop-2000.

Total RNA was converted to cDNA using the High-Capacity RNA-to-cDNA kit (Applied Biosystems, Foster City, CA) using a previously-optimized protocol [36] with slight modifications. Each reaction contained: 2 μL 20x enzyme mix, 2x RT buffer, a volume equivalent to 1 μg total RNA, and brought to a final volume of 40 μL with nuclease-free water. To check for the presence of DNA, a no-RT control, consisting of all components except the RT enzyme, was performed for each sample. Reactions were incubated in a 2720 thermocycler (Applied Biosystems, Foster City, CA) under the following conditions: 37°C for 60 min followed by heating to 95°C for 5 min. Samples were diluted 1:5 in nuclease-free water prior to use in qPCR.

### Candidate reference gene primer design and validation

Quantitative PCR assays were developed for a suite of candidate reference genes (Table 2) based on the SYBR Green chemistry [39]. For each assay, primers were designed that targeted a ca. 100–130 bp region of the coding sequence of each gene. Sequences for each gene were obtained from the annotated *Thermus* SA-01 genome (NCBI accession number CP001962) available in GenBank. Primers were designed using the Primer3 software (http://bioinfo.ut.ee/primer3/), with an optimal annealing temperature of 60°C. Primers were examined for potential secondary structures using Mfold [40]. Self and cross dimer formation was examined for each of the primer sets using the Operon oligonucleotide analysis tool (https://www.operon.com/oligos/toolkit.php), while the potential for self-complementarity was examined using an online oligonucleotide properties calculator (http://www.basic.northwestern.edu/biotools/oligocalc.html). Primers were obtained from Invitrogen (Carlsbad, CA). Primer sets were optimized over concentrations spanning 150–300 nM, with an annealing/extension temperature of 60°C. Product specificity was determined via melt curve analysis. The PCR efficiency of each assay across all culture conditions was determined using LinRegPCR [41].

**Table 2:** Function, primer sequence, PCR efficiency (PCR *E*) and melting temperature (T_M_) of candidate reference genes developed in this study.

Target	Function	Primer Sequence (5'-3')	PCR *E*	T_M_
TSC_c19900	glycosyl transferase	F: CAATGCGGAGTTTGCCTTCC	1.84	82.6
		R: GGATCGCCAAGAGTAGGAGC		
TSC_c02940	glycosyl transferase	F: GGGTGGTGGATTTGAAAGCG	1.87	78.8
		R: TTGGCGTGGCTCAAAGAAGA		
*dnaK*	chaperone	F: CTCCGTGGACCTTCTCTTGG	1.87	80.7
		R: ATGATCTGCTTGTGGGCCTC		
*gap*	glyceraldehyde-3-phosphate dehydrogenase	F: AGCCCTCATCAACGACCTTA	1.82	80.1
		R: CTCGTCATCGTAACCCACCT		
*gly*	glycosyl transferase	F: CTACGAGGAGGCCAACAACC	1.84	82.1
		R: TATGCCCACCTGCTTCACG		
*gyrA*	DNA gyrase subunit A	F: CGAGGTCATGGGCAAGTACC	1.81	84.2
		R: CCCCGTCTAAGGAGCCAAAG		
*gyrB*	DNA gyrase subunit B	F: CCCCAGTTCGAAGGTCAGAC	1.84	79.6
		R: TAGATGGTCTTGGCGATGCG		
*infA*	translation initiation factor IF-1	F: AAGGAGAAGGACACCATTCG	1.83	80.7
		R: CCGAGATGTAGGCCAGGAT		
*pfk*	6-phosphofructokinase	F: CACCATTGGCTTTGACACCG	1.81	81.7
		R: ATGAAGAAGACCCGCTCGTG		
*polA2*	DNA polymerase I	F: GGCCTTCATCGAGCGGTATT	1.83	79.5
		R: TTTCCACATAGCCCCGTTCC		
*rpoC*	RNA polymerase	F: CTCTTCAAGCCCTTCCTCCT	1.7	78.1
		R: CTGCCTTTCCAGCATCCTAC		

### Construction of external standards for absolute quantification

Absolute quantification was employed to determine the ratio of transcript to gene copies per mL as an additional means of examining reference gene stability. The expression of a subset of the candidate reference genes was also assessed via absolute quantification of the ratio of transcripts to gene copies per mL of sample. This was achieved through the creation of external calibration curves for a subset of the genes. Standards derived from PCR products were constructed for the *gyrB*, *polA2*, and *rpoC* genes, each of which occurs as a single copy within the *T*. *scotoductus* genome, and used for absolute quantification of both transcript and gene copy number. Standards were created via amplification from genomic DNA using gene-specific primers (S1 Table). Linearized PCR products were used rather than plasmid- or RNA-based standards as it has been shown that external calibration curves constructed with DNA rather than RNA serve as better models for mRNA quantification due to higher sensitivity, increased quantification range, and higher reproducibility and stability than the RNA calibration curve [42]. Additionally, plasmid-based standards have been shown to result in large overestimation compared to linear DNA in qPCR [43]. PCRs were conducted in 25 μL reaction volumes with the PCR Reagent System (Invitrogen, Carlsbad, CA) in a 2720 thermocycler (Applied Biosystems, Foster City, CA) and contained (as final concentration): 1x PCR buffer (minus Mg), 2 mM MgCl_2_, 0.25 mM each dNTP, 200 nM each forward and reverse primer, 0.5 U *Taq* polymerase, and brought to a final volume of 25 μL with nuclease-free water. Thermocycling conditions consisted of 94°C, 3 min; 35 cycles of 94°C for 45 s, 55°C for 30 s, 72°C for 45 s, and a final extension at 72°C for 7 min. PCR products were visualized by gel electrophoresis to confirm product amplification of the proper size. The resulting amplicons were purified with the QiaQuick PCR Purification kit (Qiagen), eluted in nuclease-free water, and sequenced with the gene-specific primers by GeneWiz, Inc. (Germantown, MD). Sequence identity was confirmed using the standard nucleotide BLAST function on the NCBI website. The number of copies per μL was calculated using the equation: X g μL^-1^ DNA/(PCR amplicon x 660) x 6.022 x 10^23^. Serial dilutions for each assay were prepared as described previously [36], so that the final linear range of each assay spanned from 1 x 10^2^ to 1 x 10^9^ copies. The standard curve for each assay was obtained by plotting the cycle at which fluorescence for that sample crossed the threshold value (cycle threshold, C_T_) against the calculated copy number (S1–S3 Figs). The slope, y-intercept, r^2^ value, and PCR efficiency of each assay can be found in the Supporting Information (S1 Table).

### Quantitative PCR of candidate reference genes

The Fast SYBR Green Master Mix (Applied Biosystems, Foster City, CA) was used for all reference gene qPCR assays. Each reaction contained: 10 μL 2x Fast SYBR Green master mix, 150–300 nM each forward and reverse primer, 5 ng (2 μL) template cDNA, and brought to a final volume of 20 μL with nuclease-free water. Reactions were assembled into 384-well plates using the QIAgility (Qiagen) automated pipetting system. Reactions were performed on the Vii 7 Real Time PCR System with the 384-well block format (Applied Biosystems, Foster City, CA). The following protocol was used for all assays: an initial 20 s incubation at 95°C, followed by 40 cycles of 95°C for 1 s and 60°C for 20 s, followed by a melt curve analysis of 95°C for 15 s, 60°C for 1 min, and 95°C for 15 s to determine product specificity. All qPCR reactions were performed in triplicate using Applied Biosystems MicroAmp Fast 384-well reaction plates sealed with MicroAmp optical adhesive film. No-RT controls were performed with all samples using the *rpoC* assay (S1 Data). No-template controls were also included in each amplification run to monitor for contamination. Reactions were recorded and analyzed using the Applied Biosystems ViiA 7 System software. The raw threshold cycle (C_T_) values and intra-assay variations are provided in S1 Data.

### Reference Gene Data Analysis

Three general types of culture conditions were used to determine the appropriateness of a suite of genes for use as reference genes: (1) aerobic growth in a rich (TYG) medium (10 mL culture volume); (2) anaerobic growth in basal medium supplemented with various electron donors and acceptors (10 mL culture volume); and (3) aerobic growth in an incubator versus microwave heating (60 mL culture volume). Samples were collected over a ca. 30-h time span for small-volume cultures, and 48 h for large-volume oven/microwave cultures. The expression levels of the candidate reference genes were initially determined from their C_T_ values. The BestKeeper v. 1 and NormFinder software programs were used to determine the expression stability of all 11 candidate reference genes under each of the different culture conditions and among all conditions. For data input into NormFinder, C_T_s of each gene at each time point were converted to relative quantities as described previously [36] using the equation Q = *E* ^ΔC^_T_, where ΔC_T_ = C_T_ highest abundant sample–C_T_ sample, *E* = PCR efficiency, and Q = relative quantity. It should be noted that in NormFinder, a low stability value indicates a more stable gene, while in BestKeeper, the coefficient of correlation should be as close to one as possible for a stable gene.

To determine whether instability was due to changes in gene expression or the result of multiple gene copies, indicative of polyploidy, absolute quantification was employed for a subset of the genes: *gyrB*, *polA2*, and *rpoC*. Both the number of transcripts (RNA) and gene copies (DNA) per mL was determined for each gene and used to calculate the transcript: gene copy number ratio under each of the culture conditions. The number of transcripts and gene copies per mL were calculated via external calibration curves specific for each target. Transcript or gene copy numbers from total RNA or DNA were calculated automatically from the regression lines by the ViiA 7 System software (contained in S1 Data). Gene copies per mL were calculated using an equation described previously [44], in which gene copies per mL sample = (gene copies per reaction mix x volume of DNA [μL])/ (DNA per reaction mix [μL] x mL sample used) and adjusted to account for the 1:5 initial dilution factor (volume for each sample listed in S2 Data). Each reaction utilized 2 μL of template from a 100 μL elution. Transcript copies per mL were calculated using an equation described previously [36], with a slight modification to adjust for a starting concentration of 1 μg total RNA and no initial 1:5 dilution of total RNA prior to the RT reaction. Therefore, the following equation was used to calculate transcript copies per mL: [(copies per μL per reaction mix)/(volume μL total RNA added to RT reaction/RT reaction volume μL x cDNA dilution factor] x vol total RNA eluted (μL) (S2 Data). Each reaction utilized 2 μL template from a 40 μL elution. The transcript to gene ratio was determined by dividing transcript copies per mL by gene copies per mL as described previously [36]. Ratio and C_T_ values of all three genes under all conditions were first examined for normal distribution (Shapiro-Wilkes) and equal variance (Brown-Forsythe) (SigmaPlot v.13) under each of the conditions compared (i.e. small-volume aerobic/anaerobic conditions; terminal electron acceptors; and large-volume aerobic oven/microwave incubation). If both ratio and C_T_ data passed these tests, the Pearson Product Moment correlation test was applied to examine the strength of correlation between the two. If one of the data sets failed the test for normality, the Spearman rank order correlation test was applied. T-tests or one way ANOVAs were used to test for significant differences among the ratios of biological triplicates at individual time points between culture conditions (for example, 6 h time point comparing oven versus microwave incubation) or across time under each specific condition (for example, one-way ANOVA to all ratios at all time points of cells grown aerobically in conventional oven). As with correlation analysis, if data did not pass the tests for normality and equal variance, the corresponding non-parametric tests were applied (Mann-Whitney U and Kruskall-Wallace, respectively).

## Results and Discussion

### General characteristics of candidate reference gene quantitative PCR assays

Numerous studies, across different organisms, have shown that the typical reference genes are regulated and vary under different conditions [14, 45]. Studies performed thus far have failed to identify a single gene that retains sufficient overall expression stability suitable for all contexts, and that the best candidates differ among environmental conditions [46]. Additionally, while reference gene data exist for the well-studied extremophiles *T*. *thermophilis* and *D*. *radiodurans*, minimal information exists for other extremophilic species. In the present study, 11 candidate reference genes were selected for evaluation with *T*. *scotoductus*, a thermophile shown to be capable of metal reduction and anaerobic growth in addition to aerobic growth in rich medium. Gene selection was based on a combination of parameters including their role within the cell and their use in the literature. Additionally, genes were selected from different areas of the genome (based on annotation of strain SA-01) to minimize the chance of transcriptional coupling affecting the results. For example, *rpoC* commonly serves as a reference gene in bacterial expression studies (often times without proper validation); a glycosyl hydrolase served as a reference gene for *D*. *radiodurans* in transcriptional studies upon exposure to radiation [47]; glyceraldehyde 3-phosphate dehydrogenase (*gap*) served as a reference gene in this same organism when studying DNA strand break repair [48]; *rpoC*, the chaperone *dnaK*, and genes coding for translation initiation factors were used in prior expression studies concerning *T*. *thermophilis* bacteriophage [49]; and *rpoC* and the gyrase subunit A (*gyrA*) were employed in transcriptome studies with the extremophile *Acidithiobacillus ferrooxidans* [28]. *gyrA* has also been evaluated as a reference gene with other bacterial species, with conflicting results (i.e. inappropriate for use with *Staphylococcus aureus* [50], appropriate with the plant pathogen *Pectobacterium atrosepticum* [51]).

**Fig 1:**
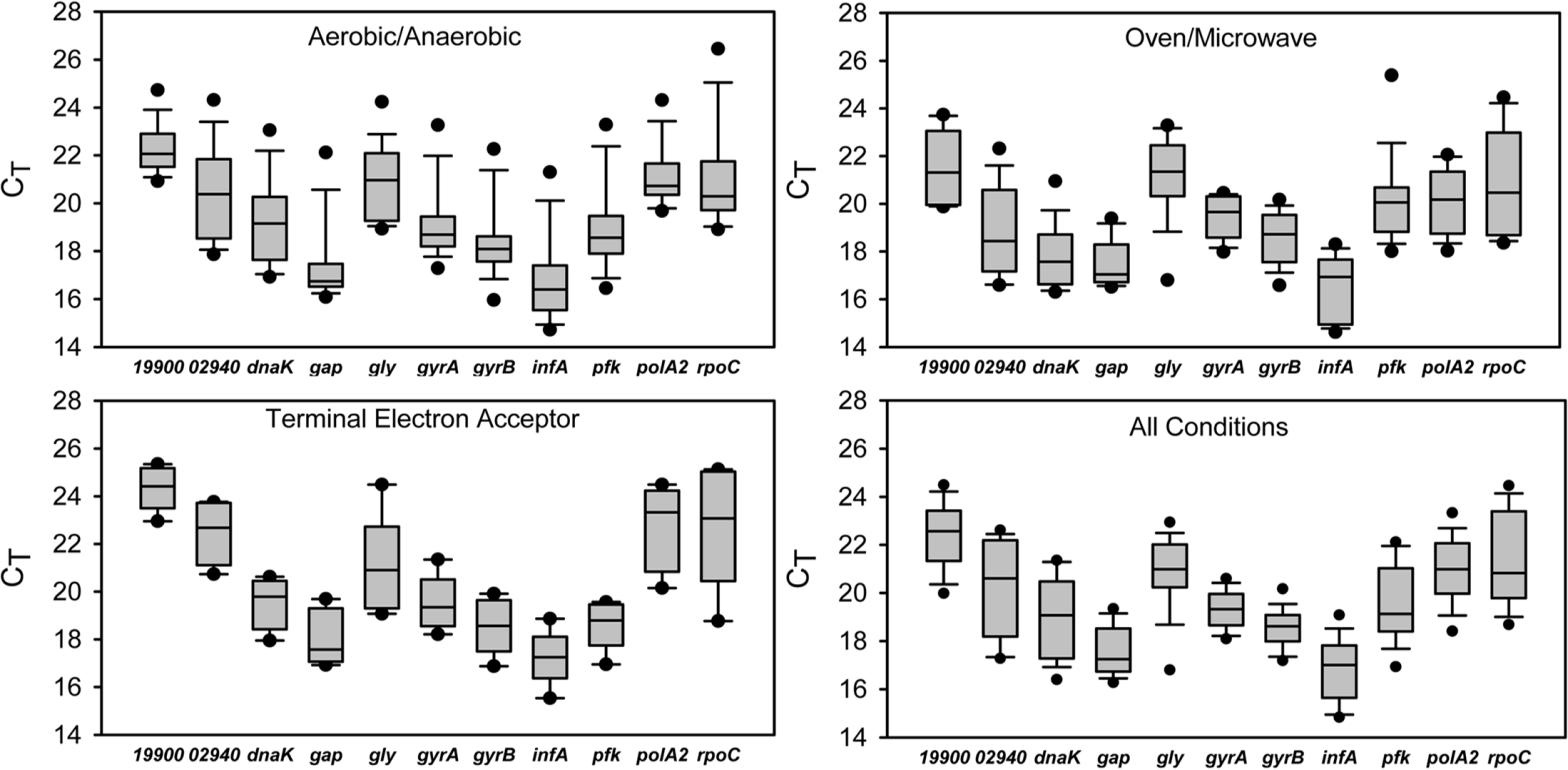
Boxplot of C_T_ values of candidate reference genes under all culture conditions. A line across the box depicts the median. The box indicates the 25^th^ and 75^th^ percentiles. Whiskers represent the 10^th^ and 90^th^ percentiles. Filled circles represent 5^th^ and 95^th^ percentiles.

The compliance of the RT-qPCR assays with the MIQE (Minimum Information for Publication of Quantitative Real-Time PCR Experiments [52]) guidelines is shown in the MIQE checklist (S2 Table). All assays were specific for their intended gene target, as indicated by a single peak in the melt curve analysis (S2 Fig) and possessed similar PCR efficiencies (Table 2). The average C_T_ values, when assimilating the data from all 11 genes, ranged from ca. 16.8–22.3 under anaerobic/aerobic growth over time; 17.2–24.4 when comparing anaerobic growth using different terminal electron acceptors; and 16.5–21.6 under different heating methods (i.e. oven or microwave incubation), and 16.7–22.3 under all conditions combined (Fig 1 and Tables 3–6).

**Table 3:** Descriptive statistics and stability values of candidate reference genes under anaerobic (glucose, lactate) and aerobic (TYG) growth over time (*n* = 24).

Target	*dnaK*	*gap*	*gly*	*gyrA*	*gyrB*	*infA*	*pfk*	*polA2*	20940	19900	*rpoC*
geo Mean [C_T_]	19.12	17.30	20.90	18.99	18.28	16.67	18.90	21.05	20.26	22.27	20.92
ar Mean [C_T_]	19.19	17.36	20.96	19.04	18.33	16.75	18.97	21.08	20.34	22.29	21.01
min [C_T_]	16.91	16.04	18.90	17.14	15.67	14.68	16.46	19.68	17.82	20.90	18.90
max [C_T_]	23.18	22.41	24.68	23.30	22.38	21.42	23.28	24.57	24.52	24.88	26.55
SD [±C_T_]	1.38	1.02	1.34	0.99	1.00	1.28	1.27	0.90	1.54	0.80	1.54
CV [%C_T_]	7.21	5.90	6.41	5.22	5.46	7.62	6.68	4.28	7.55	3.59	7.32
coeff. of corr. [r]	0.91	0.98	0.57	0.91	0.89	0.93	0.90	0.98	0.89	0.83	0.95
p-value	0.00	0.00	0.00	0.00	0.00	0.00	0.00	0.00	0.00	0.00	0.00
NF Medium	0.466	0.196	0.781	0.373	0.394	0.353	0.447	0.137	0.533	0.401	0.405
NF Time	0.178	0.072	0.332	0.143	0.166	0.117	0.169	0.059	0.224	0.174	0.136
NF Medium and Time	0.492	0.290	0.765	0.451	0.486	0.434	0.498	0.208	0.533	0.501	0.430
NF No Groupings	0.490	0.197	0.917	0.393	0.461	0.426	0.469	0.116	0.621	0.504	0.388

SD = standard deviation, NF = NormFinder

**Table 4:** Descriptive statistics and stability values of candidate reference genes during anaerobic growth with different terminal electron acceptors (*n* = 5).

Target	*dnaK*	*gap*	*gly*	*gyrA*	*gyrB*	*infA*	*pfk*	*polA2*	*rpoC*	*19900*	*20940*
geo Mean [C_T_]	19.49	18.03	20.91	19.47	18.54	17.21	18.62	22.64	22.68	24.34	22.44
ar Mean [C_T_]	19.51	18.06	20.99	19.49	18.57	17.24	18.64	22.70	22.81	24.36	22.47
min [C_T_]	17.95	16.93	19.07	18.22	16.88	15.54	16.95	20.15	18.77	22.96	20.73
max [C_T_]	20.62	19.69	24.49	21.34	19.91	18.86	19.56	24.49	25.13	25.35	23.77
SD [± C_T_]	0.87	0.99	1.40	0.81	0.86	0.70	0.72	1.49	1.89	0.68	1.09
CV [% C_T_]	4.44	5.49	6.67	4.16	4.62	4.04	3.85	6.56	8.29	2.81	4.85
coeff. of corr. [*r*]	0.87	0.82	0.66	0.87	0.91	0.85	0.69	0.97	0.89	0.92	0.99
NF	0.454	0.510	0.939	0.481	0.321	0.451	0.590	0.519	0.819	0.287	0.224

SD = standard deviation, NF = NormFinder

**Table 5:** Descriptive statistics and stability values of candidate reference genes under different heating methods (oven or microwave) (*n* = 16).

Target	*dnaK*	*gap*	*gly*	*gyrA*	*gyrB*	*infA*	*pfk*	*polA2*	*20940*	*19900*	*rpoC*
geo Mean [C_T_]	17.76	17.46	21.18	19.40	18.56	16.49	20.00	20.03	18.84	21.53	20.82
ar Mean [C_T_]	17.80	17.48	21.24	19.42	18.59	16.54	20.06	20.07	18.93	21.58	20.92
min [C_T_]	16.30	16.52	16.80	17.98	16.59	14.63	18.01	18.03	16.59	19.88	18.36
max [C_T_]	20.95	19.38	23.28	20.46	20.17	18.30	25.39	22.05	22.31	23.74	24.46
SD [± C_T_]	1.03	0.80	1.15	0.75	0.84	1.09	1.13	1.08	1.67	1.41	1.83
CV [% C_T_]	5.80	4.59	5.40	3.85	4.52	6.61	5.63	5.38	8.84	6.51	8.74
coeff. of corr. [r]	0.88	0.77	0.08	0.85	0.86	0.93	0.75	0.98	0.88	0.91	0.96
p-value	0.00	0.00	0.78	0.00	0.00	0.00	0.00	0.00	0.00	0.00	0.00
NF Heating	0.225	0.351	0.740	0.237	0.311	0.281	0.302	0.071	0.416	0.220	0.282
NF Time	0.274	0.354	0.823	0.380	0.334	0.195	0.437	0.115	0.579	0.376	0.475
NF Heating and Time	0.207	0.491	0.625	0.373	0.330	0.311	0.468	0.138	0.531	0.436	0.531

SD = standard deviation, NF = NormFinder

**Table 6:** Descriptive statistics and stability values of candidate reference genes across all conditions and time points (*n* = 45).

Target	*dnaK*	*gap*	*gly*	*gyrA*	*gyrB*	*infA*	*pfk*	*polA2*	*20940*	*19900*	*rpoC*
geo Mean [C_T_]	18.66	17.44	21.00	19.19	18.40	16.66	19.25	20.85	19.97	22.22	21.07
ar Mean [C_T_]	18.73	17.48	21.06	19.23	18.45	16.73	19.32	20.90	20.08	22.27	21.18
min [C_T_]	16.30	16.04	16.80	17.14	15.67	14.63	16.46	18.03	16.59	19.88	18.36
max [C_T_]	23.18	22.41	24.68	23.30	22.38	21.42	25.39	24.57	24.52	25.35	26.55
SD [± C_T_]	1.34	0.96	1.30	0.93	0.95	1.17	1.26	1.14	1.75	1.19	1.80
CV [% C_T_]	7.17	5.51	6.18	4.86	5.17	6.99	6.54	5.48	8.73	5.33	8.50
coeff. of corr. [r]	0.87	0.91	0.41	0.86	0.86	0.92	0.72	0.91	0.86	0.79	0.93
p-value	0.00	0.00	0.01	0.00	0.00	0.00	0.00	0.00	0.00	0.00	0.00
NF	0.517	0.266	0.873	0.432	0.470	0.324	0.719	0.377	0.726	0.518	0.503

SD = standard deviation, NF = NormFinder

### Reference Gene Stability during Aerobic and Anaerobic Growth Conditions

The expression stability of candidate reference genes was initially evaluated based on calculated variations (SD, standard deviation of the C_T_, and CV, the coefficient of variance expressed as a percentage on the C_T_ level) using BestKeeper. In general, a lower SD equates to greater stability, and any gene with the SD > 1.00 is considered inconsistent [37]. In addition to stability (SD and CV) values, additional key parameters include the Pearson correlation coefficient (*r)*, derived from pair-wise correlation analyses that estimate inter-gene relationships among all reference genes, and their associated probability (*p*) values. The comprehensive results of the BestKeeper analysis are provided in S3 Data. The candidate reference genes were then ranked to determine the best combination of genes under each condition and across all conditions using the program NormFinder. One of the valuable functions of this program, in addition to assessing overall expression variation, is that the samples can be assigned to different groups, so that the intra- and intergroup expression variation of the candidate genes can be assessed. This allows for the identification of specific conditions under which each gene displays greatest variance. When comparing reference gene expression across time among aerobic growth in a rich medium (TYG) and anaerobic growth in basal medium supplemented with glucose or lactate as the carbon source (with nitrate as terminal electron acceptor in both), stability values as determined via BestKeeper ranged from 0.80–1.54. The glycosyl hydrolase TSC_c19900 (hereafter referred to as “19900”) ranked as the most stable across time and all conditions, followed by *polA2* (0.90), *gyrA* (0.99), *gyrB* (1.00), and *gap* (1.02). These were the only genes with a SD ≤ 1.00. A gene commonly employed as reference gene, *rpoC*, was determined to be the least stable among these conditions (SD = 1.54), along with the glycosyl hydrolase TSC_c02940 (hereafter referred to as “02940”) (SD = 1.54) (Table 3). Growth conditions were analyzed three different ways with NormFinder: (1) groups assigned by medium type (glucose/nitrate, lactate/nitrate, or TYG); (2) groups assigned by time (early, mid, and late growth); and (3) groups assigned by both medium and time (Table 3). The most appropriate gene when samples were assigned by medium type was *polA2* (0.137), with the best combination of genes identified as *gap* and *polA2* (0.144) (S4 Data). With regards to NormFinder analysis, potential reference genes should display an inter-group variance as close to zero as possible [38]. However, it is important to note that the simultaneous use of genes with positive and negative inter-group values would yield lower stability values than sets involving genes with the same sign due to compensating effects [53]. All genes displayed positive and negative inter-group values, with a summation totaling zero (S3 Table). When samples were grouped by time, *polA2* was also identified as the most stable (0.059), with the best combination being *gap* and *polA2* (0.047) (S4 Data). All genes also displayed positive and negative inter-group values by this grouping mechanism, with summation of all three variables totaling zero (S3 Table). *PolA2* was also identified as the gene with the lowest stability value (0.208) when grouping by both time and medium, with the best combination identified as *gap* and *polA2* (0.195) (S4 Data). *Gly* was identified as the least stable gene. As with the other groupings, grouping by both time and medium resulted in a summation of zero when totaling the intergroup values for all 9 variables (S3 Table). Analysis with no grouping also identified *polA2* as most stable (0.116), followed by *gap* (0.197). With samples not defined by groups, NormFinder identified *gly* as the least stable (0.917), followed by 02940.

### Reference Gene Stability during Anaerobic Growth with Varied Electron Acceptors

Under conditions of anaerobic growth with different terminal electron acceptors, the most stable genes as determined via BestKeeper were 19900, *infA*, and *pfk* (Table 4). All three displayed very low and very similar SD values of 0.68, 0.70, and 0.72, respectively. As the PCR efficiencies of these assays are also very similar (1.84, 1.83, and 1.81, respectively [Table 2]), any of these three could be used alone or in combination as appropriate reference gene in the short-term (i.e. 24 h growth) expression analyses under anaerobic growth with different terminal electron acceptors. Following these three genes were the gyrase subunits, *gyrA* (0.81) and *gyrB* (0.86). Similar to the results obtained when comparing aerobic and anaerobic growth over time, *rpoC* would serve as the least appropriate reference gene under these conditions as well, as it was the least stable by a relatively large margin (SD = 1.89) (Table 4) among the set. NormFinder identified 02940 as having the lowest stability value (0.224), with the best combination being 02940 and 19900 (0.225) (S4 Data). In agreement with the BestKeeper analysis, these were followed by *gyrB* and *infA*. All genes displayed a cumulative intergroup variation of zero, as all displayed both a positive and negative inter-group value. When considering the intragroup variation of cells incubated with iron as the terminal electron acceptor, *gap*, 02940, *pfk*, and *dnaK* displayed the lowest values, indicative of stability among samples under this condition, while 19900, *infA*, *gyrB*, and *polA2* exhibited the greatest stability among samples grown in the controls in which nitrate served as the terminal electron acceptor (S4 Table).

### Reference Gene Stability during Oven or Microwave Incubation

When comparing heating methods (oven versus microwave) at 65°C, BestKeeper analysis identified *gyrA* (0.75), *gap* (0.80) and *gyrB* (0.84) as the most stable genes. The SD of multiple genes were equal to or slightly greater than the recommended cut-off of 1.00 (*dnaK*, *infA*, *polA2*) (Table 5). As with the results obtained under varying anaerobic growth conditions as well as aerobic growth, *rpoC* was the least stable gene (SD = 1.83), along with 02940 (Table 5). Oven and microwave-grown samples were then analyzed by different groupings in NormFinder: (1) heating method (oven or microwave) (2) time (3) both variables combined and (4) no groupings. The most appropriate reference gene based on heating method was *polA2* (0.071), with *polA2* and 19900 as the best combination (0.120) (Table 5 and S4 Data). In accordance with these results, *polA2* displayed the lowest intragroup variation among all assays under either heating method (MW = 0.001, oven = 0.002), followed by *infA* (0.003), *dnaK* (0.014), and 19900 (0.076) under oven heating, and *gyrB* (0.019), *gyrA* (0.020), and *infA* (0.056) under microwave heating (S5 Table). *PolA2* (0.115) was also the most stable gene across time, with *infA* and *polA2* as best combination (0.088) (S4 Data). Supporting these individual grouping analyses, *polA2* was also the most stable based on combined heating method and time (0.138), with *polA2* and *dnaK* as best combination (0.141) (S4 Data). The intra- and intergroup values when grouping by heating type; time; and a combination of heating type and time can be found in the Supplementary Information S5 Table.

### Reference gene stability across all conditions

Assimilating the results of both BestKeeper and NormFinder under the various culture conditions, different genes were identified as the most stable under different conditions. While *gyrB* and 19900 both ranked among the top four in two out of the three treatment comparisons (*gyrB*, aerobic versus anaerobic growth and oven versus microwave heating; 19900, aerobic versus anaerobic growth, anaerobic growth comparing terminal electron acceptors) there was not a single gene that was consistently identified as the most stable among all conditions. Therefore, in order to accurately identify the most stable genes under all conditions, all data were combined and analyzed with both BestKeeper and NormFinder. The results of BestKeeper revealed that 3 of the 11 genes displayed acceptable stability across all conditions (reflected by a SD <1.00): *gap*, *gyrA*, and *gyrB;* additionally, *infA*, *polA2*, and 19900 were just slightly above the cut-off of 1 (all <1.20) (Table 6). Conversely, the gene most frequently used for normalization, *rpoC*, displayed the greatest variance. The most appropriate gene for use across all conditions as identified via NormFinder was *gap*, with the best combination consisting of *gap* and *infA*, with a combined stability value of 0.206 (Table 6 and S4 Data). *PolA2*, *gyrA*, and *gyrB* were the next most-stable genes among the 11 according to NormFinder. In keeping with the positive and negative values obtained for the intergroup variation and the resulting summation of zero, all genes also displayed a summation of zero when combining all conditions (S6 Table).

### Absolute quantification of transcript and gene copy numbers

Other bacterial species closely related to *T*. *scotoductus* (*T*. *thermophilis*, *D*. *radiodurans*) have been shown to exhibit polyploidy. Additionally, growth stage and/or environmental factors have been shown to stimulate polyploidy in bacterial species not typically associated with this trait (i.e. *E*. *coli*, *P*. *aeruginosa*). Based on this, expression fluctuations in a subset of the potential reference genes were examined with absolute quantification in order to investigate whether fluctuations in C_T_s were indicative of changes in transcript abundance, or whether C_T_ fluctuations were an indication of changes in gene copy number. This would be the case if cells were transitioning between mono- and polyploidy. The three genes were selected based on a combination of factors, as they play different roles within the cell and ranged in their stability values under the various growth conditions.

**Fig 2:**
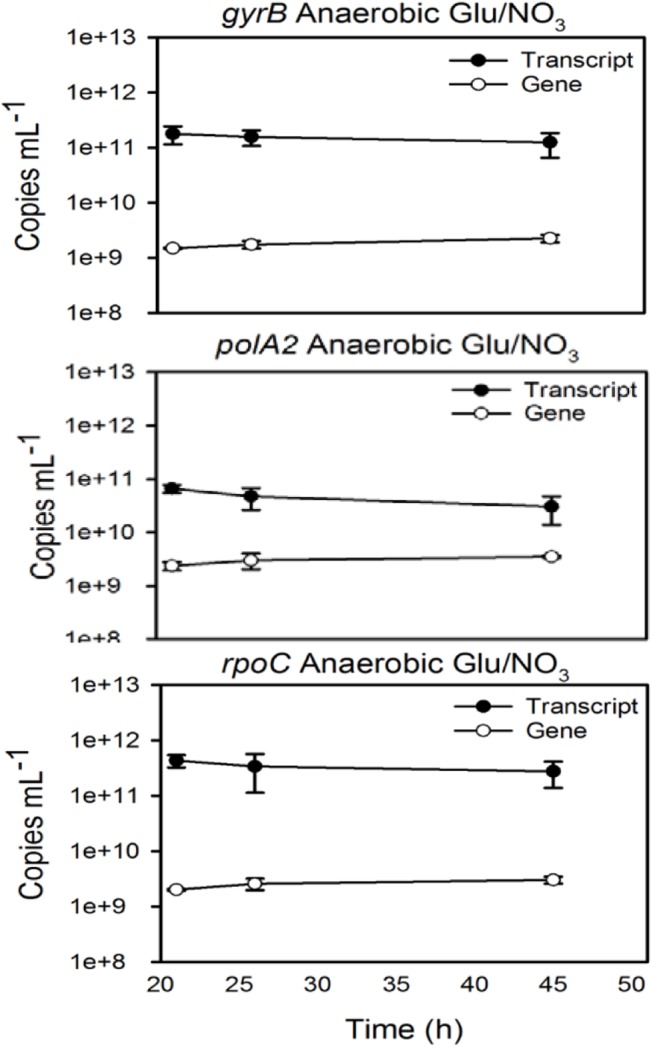
Representative figure of data used to obtain transcript-to-gene ratios for *gyrB*, *polA2*, and *rpoC*. Samples were collected at multiple time points from anaerobic cultures supplemented with glucose as the carbon source and nitrate as the terminal electron acceptor. Transcript and gene copies per mL of sample were calculated via absolute quantification using external calibration curves. Error bars represent the standard deviation derived from triplicate qPCRs from three biological replicates.

**Fig 3:**
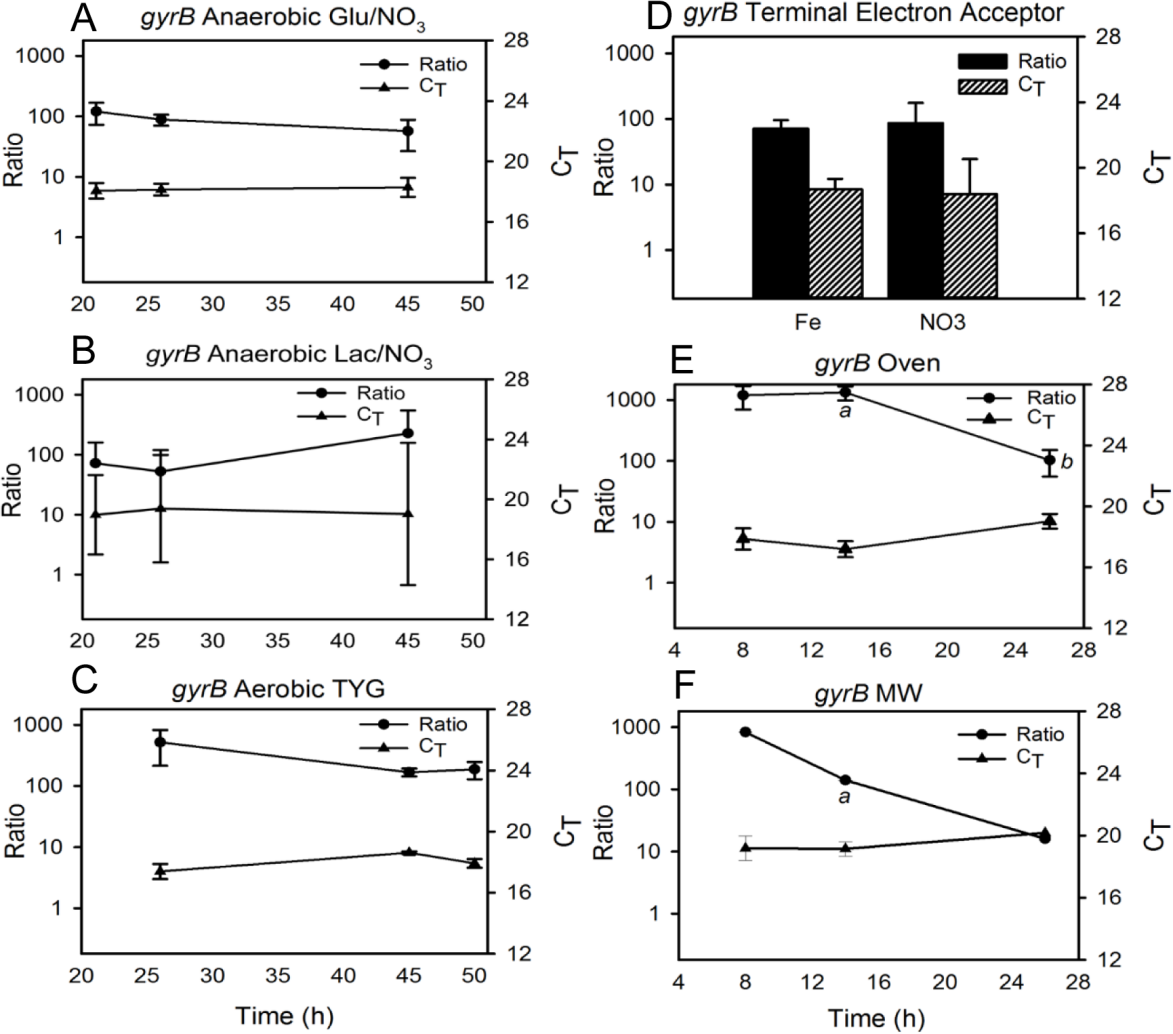
Correlation between *gyrB* transcript C_T_ values and gene expression as derived from the transcript-to-gene copy number ratios. A standard curve was constructed and used to calculate the transcript copies per mL of sample and gene copies per mL of sample (“transcript-to-gene ratio”) under the different culture conditions described in the text. Ratio and C_**T**_ values obtained for *gyrB* under: anaerobic growth with (A) glucose or (B) lactate as carbon source compared to (C) small-volume aerobic growth in TYG; (D) anaerobic growth with glucose as carbon source and nitrate or iron as terminal electron acceptor; and aerobic growth in TYG using (E) oven or (F) microwave heating. Glu = glucose, Lac = lactate, MW = microwave heating. a = indicates statistically significant difference (p < 0.05) among different culture conditions at specific time points; b = indicates statistically significant difference (p < 0.05) across time under one culture condition.

**Fig 4:**
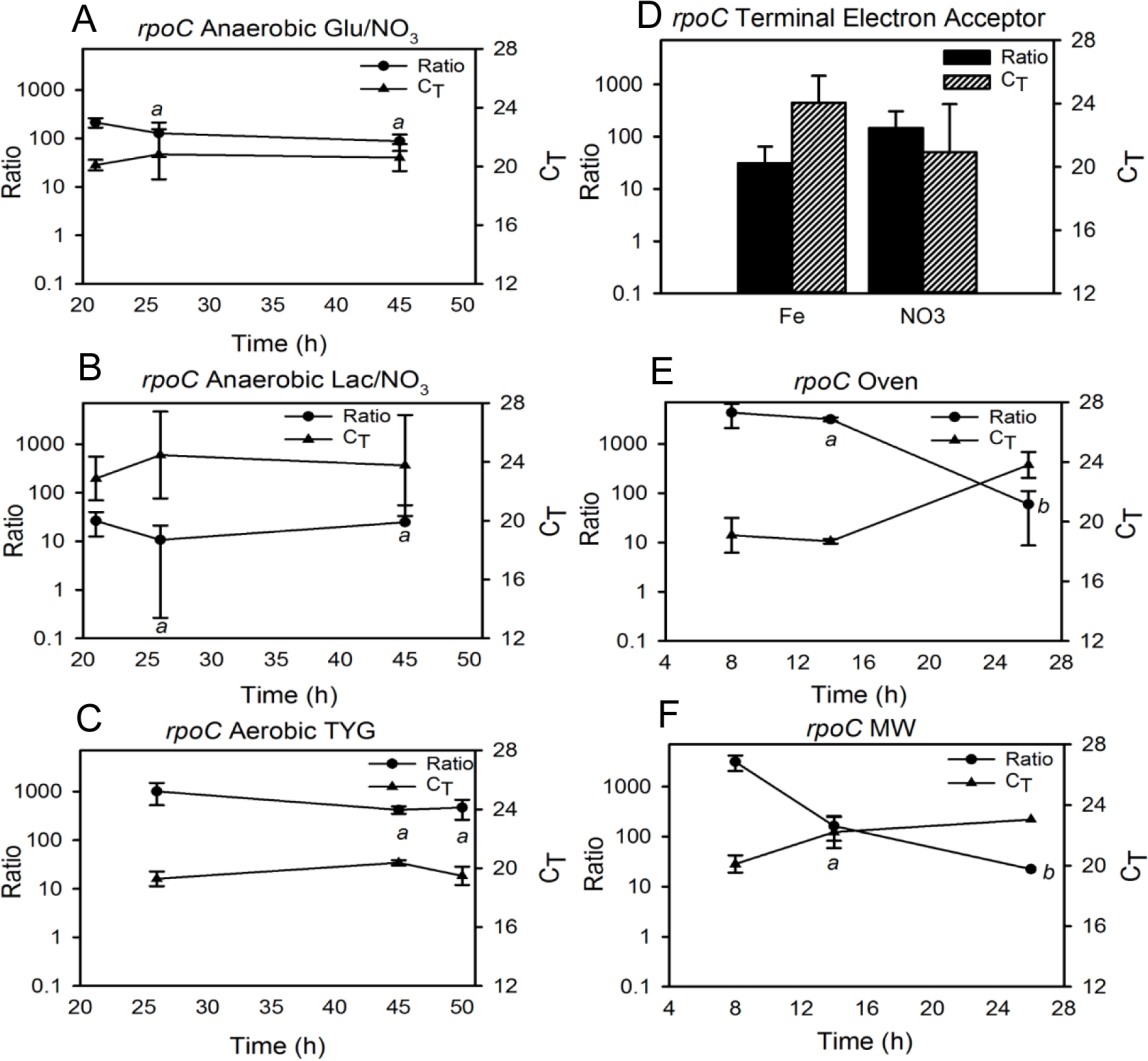
Correlation between *rpoC* transcript C_T_ values and gene expression as derived from the transcript-to-gene copy number ratios. A standard curve was constructed and used to calculate the transcript copies per mL of sample and gene copies per mL of sample (“transcript-to-gene ratio”) under the different culture conditions described in the text. Ratio and C_**T**_ values obtained for *rpoC* under: anaerobic growth with (A) glucose or (B) lactate as carbon source compared to (C) small-volume aerobic growth in TYG; (D) anaerobic growth with glucose as carbon source and nitrate or iron as terminal electron acceptor; and aerobic growth in TYG using (E) oven or (F) microwave heating. Glu = glucose, Lac = lactate, MW = microwave heating. a = indicates statistically significant difference (p < 0.05) among different culture conditions at specific time points; b = indicates statistically significant difference (p < 0.05) across time under one culture condition.

**Fig 5:**
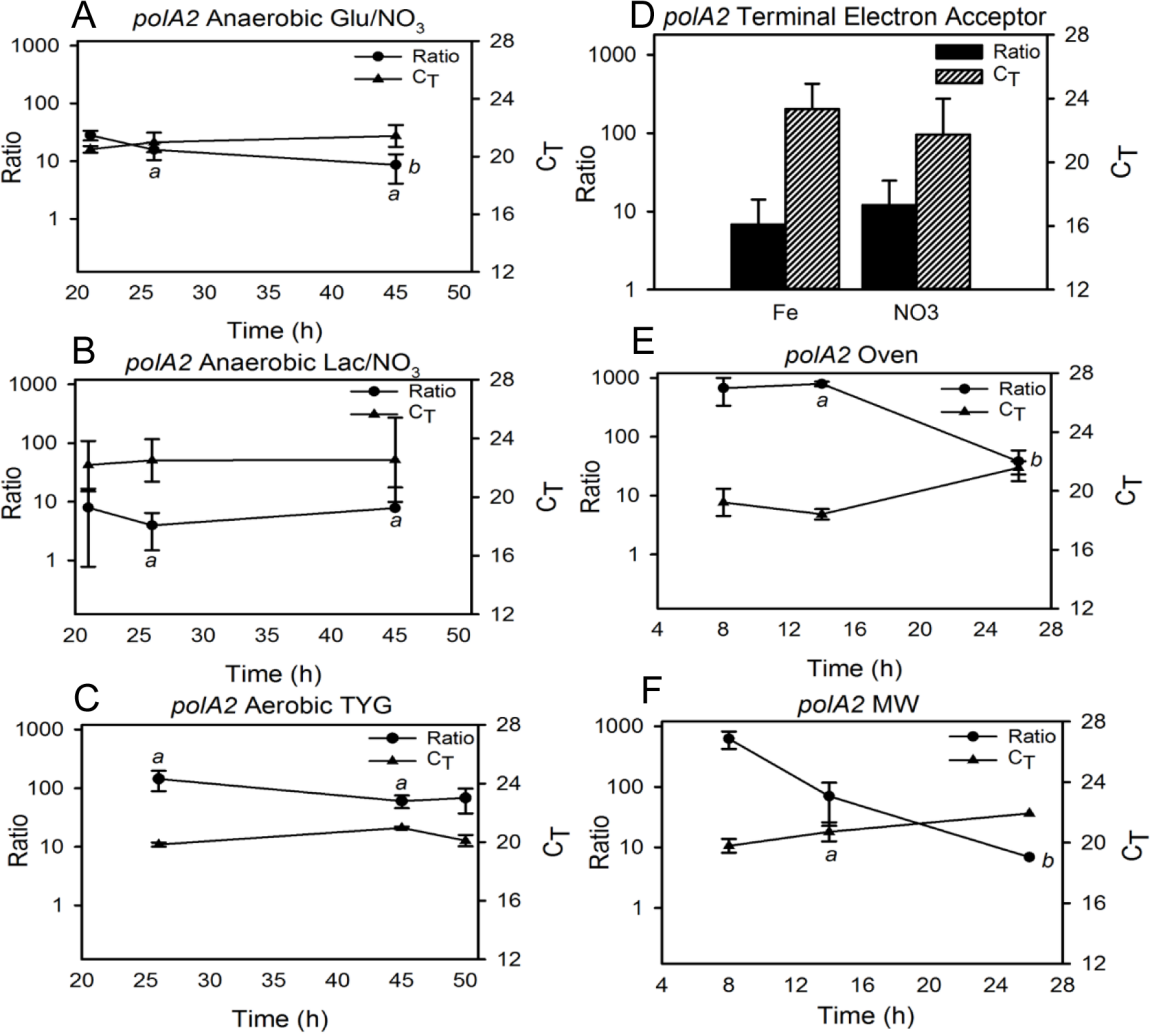
Correlation between *polA2* transcript C_T_ values and gene expression as derived from the transcript-to-gene copy number ratios. A standard curve was constructed and used to calculate the transcript copies per mL of sample and gene copies per mL of sample (“transcript-to-gene ratio”) under the different culture conditions described in the text. Ratio and C_**T**_ values obtained for *polA2* under: anaerobic growth with (A) glucose or (B) lactate as carbon source compared to (C) small-volume aerobic growth in TYG; (D) anaerobic growth with glucose as carbon source and nitrate or iron as terminal electron acceptor; and aerobic growth in TYG using (E) oven or (F) microwave heating. Glu = glucose, Lac = lactate, MW = microwave heating. a = indicates statistically significant difference (p < 0.05) among different culture conditions at specific time points; b = indicates statistically significant difference (p < 0.05) across time under one culture condition.

External, PCR-amplicon-based standards were created and used to calculate both transcript and gene copy numbers per mL of sample in order to determine the ratio of transcript copies to gene copies under the various culture conditions (representative image in Fig 2, plots of transcript and gene copy number for all genes under all conditions S5–S13 Figs). This transcript: gene copy number ratio indicated whether C_T_ fluctuations were due to changes in transcript abundance, indicative of changes in gene expression, or changes in gene copy number, such as would occur during mono/polyploidy transitions. As described in a previous study, the Pearson Product Correlation test was applied to the ratio and corresponding C_T_ of each time point for each gene to test the hypothesis that a negative correlation exists between ratio and C_T_ [36]. With the exception of *gyrB* across small-volume aerobic/anaerobic conditions (Table 7, “*gyrB* Growth”), statistically significant negative correlations were found to exist between the transcript: gene ratio and C_T_ values among the three genes under all conditions (Table 7). These correlations provide strong evidence that C_T_ changes are an accurate depiction of transcript copy number, and not gene copy number, fluctuations. Plotting both the transcript: gene copy number ratio and C_T_ values over time illustrates that in general, C_T_ changes reflect fluxes in transcript copy number (Figs 3–5). For example, when grown aerobically in an oven, the *gyrB* C_T_ and ratio values showed an inverse trend over time (Fig 3E). If this were due to changes in gene (DNA) copy number, the ratio pattern would mirror that of the C_T_s. However, an increase in ratio is strongly indicative of an increase in transcript abundance. These data demonstrate that even with the potential for polyploidy, relative quantification is applicable for measuring gene expression changes in extremophiles.

**Table 7:** Pearson Product Correlation values obtained for ratio and C_T_ of *gyrB*, *polA2*, and *rpoC* under each condition.

Target/Condition	r	p-value
*gyrB* Growth	-0.52	0.14*
*rpoC* Growth	-0.97	0.00*
*polA2* Growth	-0.97	0.00*
*gyrB* e-	-1.00	0.00
*rpoC* e-	-1.00	0.02
*polA2* e-	-0.98	0.00
*rpoC* O/MW	-0.94	0.00
*polA2* O/MW	-0.95	0.00
*gyrB* O/MW	-0.87	0.03

* indicates Spearman rank value

e- = terminal electron acceptor experiments

O/MW = oven v microwave heating experiments

### Absolute Quantification to Identify Specific Fluctuations

The data obtained via absolute quantification was then used to identify the specific conditions and times under which gene expression fluctuated. Gene copy number was similar among the three genes under each condition (representative image of transcript and gene copy numbers per mL for each of the three genes is shown in Fig 2, transcript and gene copy numbers per mL for all three genes under all conditions shown in S5–S13 Figs), corroborating genome annotation analysis that showed each gene existing as a single copy within the genome. PCR efficiencies (PCR *E*) were similar among the three assays (Table 2) and so should result in similar gene copy numbers under a specific treatment barring unusual changes in cell physiology. Therefore, ratio fluctuations could be attributed to changes in transcript copy numbers.

There was no significant difference among *gyrB* ratios at either 26 or 45 h of growth under aerobic or anaerobic cultures, nor was there any significant difference in ratios across time under any of these three conditions (Fig 3A–3C). These results corroborated with those obtained with BestKeeper based solely on C_T_ values. There was also no significant difference between *gyrB* ratios when cells were grown anaerobically with iron or nitrate as the terminal electron acceptor (Fig 3D). In comparing the ratios of *gyrB* for cells grown in an oven compared to a microwave, there was no significant difference at 8 h, but there was at 14 h (t-test, p = 0.00) (Fig 3E and 3F); additionally, there was no significant difference in *gyrB* ratios over time during microwave incubation (one-way ANOVA, p = 0.06), but there was during oven incubation (one-way ANOVA, p = 0.00).

Ratios for *rpoC* were also found to differ significantly at multiple time points when comparing aerobic (TYG) and anaerobic (with glucose or lactate as carbon source in a minimal medium) growth (26 h, one-way ANOVA, p = 0.03, 45 h one-way ANOVA, p < 0.00) (Fig 4A–4C). Additionally, there was not a significant difference in *rpoC* ratios between cells grown with different terminal electron acceptors (Fig 4D). Ratios for *rpoC* followed a similar trend to that of *gyrB* for oven and microwave growth, with a significant difference at 14 h (t-test, p = 0.00) but not 8 h (Fig 4E and 4F). There was also a significant difference in *rpoC* ratios across time when cells were grown in an oven (one-way ANOVA, p = 0.02) or with microwave heating (Kruskall-Wallace, p = 0.04).

*PolA2* differed significantly at both 26 h and 45 h when comparing aerobic and anaerobic growth (one-way ANOVA, p = 0.01, p = 0.00, respectively), and also across time when grown anaerobically in minimal medium with glucose as the carbon source (one-way ANOVA, p = 0.01) (Fig 5A–5C). However, there was no significant difference across time when cells were grown anaerobically with lactate as the sole carbon source, or aerobically in TYG (Fig 5B and 5C). There was also no significant difference in the ratios when cells were grown anaerobically with different electron acceptors (Fig 5D). As with *gyrB* and *rpoC*, *polA2* ratios were not significantly different after 8 h growth in oven or MW, yet were after 14 h (t-test, p = 0.00). *PolA2* ratios were also found to differ significantly across time under both oven and microwave incubation (one-way ANOVA, p = 0.01; Kruskall-Wallace, p = 0.04, respectively) (Fig 5E and 5F).

## Conclusions

In this study, we designed and validated assays for a suite of reference genes for use in *T*. *scotoductus* expression studies under multiple culture conditions. Based on their unique biomolecular properties that have allowed them to adapt to extreme environments, the question arises as to whether mesophilic reference genes are suitable for use with extremophiles. Under aerobic growth and anaerobic growth with different electron donors and nitrate as the terminal electron acceptor, 19900, *polA2*, *gyrA*, and *gyrB* ranked as the most stable genes. Under conditions of anaerobic growth with varied electron acceptors, 19900, *infA*, *pfk*, *gyrA* and *gyrB* were the most stable. Three genes displayed a SD < 1.00 under aerobic growth using different heating methods: *gyrA*, *gap*, and *gyrB*. Overall, the results demonstrate that when assimilating all culture conditions, *gap*, *gyrA*, and *gyrB* are the most stable (SD < 1.00) and thus the most appropriate if using a combination of conditions. The consistent stability of *gyrA* is similar to the results obtained in reference gene studies with mesophiles. We also demonstrated that *rpoC* does not serve as a reliable reference gene in thermophiles, due to its expression instability across all culture conditions. Absolute quantification with three of the genes demonstrated that even with the potential for polyploidy, relative quantification is applicable for use in RT-qPCR expression studies with extremophiles.

## Supporting Information

S1 Data Raw C_T_ values and intra-assay variations for 11 reference genes assessed in this study. For genes (*gyrB*, *polA2*, *rpoC*) assessed via absolute and relative quantification, “DNA” refers to qPCRs in which DNA served as template and was used to calculate gene copies per mL; “RNA” refers to qPCRs in which cDNA served as template and was used to calculate transcript copies per mL.
(XLSX)

S2 Data Sample name, description and values used for transcript and copy number per mL calculations. Data include: total RNA concentration, volume added to RT reaction, and volume of sample collected for DNA extraction.
(XLSX)

S3 Data Comprehensive output of BestKeeper analysis. (XLSX)

S4 Data NormFinder stability values for best combination of genes under each culture condition. (XLSX)

S1 Fig Standard curve of *gyrB* assay. A 700 base pair region of the gyrase subunit B gene from the *Thermus scotoductus* genome was amplified using gene-specific primers. The resulting PCR product was purified and used in the construction of an external calibration curve. The number of copies per μL was calculated using the equation: X g μL^-1^ DNA/(PCR amplicon length x 660) x 6.022 x 10^23^. Serial 10-fold dilutions were prepared that spanned eight orders of magnitude ranging from 1 x 10^2^ to 1 x 10^9^ copies. The standard curve was determined by plotting the log of the calculated copy number against the cycle at which fluorescence for that sample crossed the threshold cycle. The slope, y-intercept, r^2^ value, and PCR efficiency of the *gyrB* assay is provided in S1 Table.
(TIF)

S2 Fig Standard curve of the *polA2* assay, constructed as described for the *gyrB* assay. (TIF)

S3 Fig Standard curve of the *rpoC* assay, constructed as described for the *gyrB* assay. (TIF)

S4 Fig Melt curve profiles of the 11 reference genes evaluated in this study. (TIF)

S5 Fig *gyrB* transcript and gene copies over time comparing anaerobic versus aerobic culture conditions. *T*. *scotoductus* cultures were grown anaerobically in a basal medium with lactate as the carbon source and nitrate as the terminal electron acceptor (top panel) or aerobically in TYG medium (bottom panel). Transcript and gene copies per mL of sample were calculated via absolute quantification using external calibration curves as described in the text. Error bars represent the standard deviation of triplicate qPCRs from three biological replicates.
(TIF)

S6 Fig *gyrB* transcript and gene copies over time comparing oven versus microwave heating. *T*. *scotoductus* cultures were grown aerobically in TYG using oven (top panel) or microwave (bottom panel) heating. Transcript and gene copies per mL of sample were calculated via absolute quantification using external calibration curves as described in the text. Error bars represent the standard deviation of triplicate qPCRs from three biological replicates.
(TIF)

S7 Fig *gyrB* transcript and gene copies after 24 h comparing terminal electron acceptors. *T*. *scotoductus* was grown anaerobically in basal medium with glucose as the carbon source and iron (Fe) or nitrate (NO_3_) as the terminal electron acceptor. Transcript and gene copies per mL of sample were calculated via absolute quantification using external calibration curves as described in the text. Error bars represent the standard deviation of triplicate qPCRs from three biological replicates.
(TIF)

S8 Fig *rpoC* transcript and gene copies over time comparing anaerobic versus aerobic culture conditions. *T*. *scotoductus* cultures were grown anaerobically in a basal medium with lactate as the carbon source and nitrate as the terminal electron acceptor (top panel) or aerobically in TYG medium (bottom panel). Transcript and gene copies per mL of sample were calculated via absolute quantification using external calibration curves as described in the text. Error bars represent the standard deviation of triplicate qPCRs from three biological replicates.
(TIF)

S9 Fig *rpoC* transcript and gene copies over time comparing oven versus microwave heating. *T*. *scotoductus* cultures were grown aerobically in TYG using oven (top panel) or microwave (bottom panel) heating. Transcript and gene copies per mL of sample were calculated via absolute quantification using external calibration curves as described in the text. Error bars represent the standard deviation of triplicate qPCRs from three biological replicates.
(TIF)

S10 Fig *rpoC* transcript and gene copies after 24 h comparing terminal electron acceptors. *T*. *scotoductus* was grown anaerobically in basal medium with glucose as the carbon source and iron (Fe) or nitrate (NO_3_) as the terminal electron acceptor. Transcript and gene copies per mL of sample were calculated via absolute quantification using external calibration curves as described in the text. Error bars represent the standard deviation of triplicate qPCRs from three biological replicates.
(TIF)

S11 Fig *polA2* transcript and gene copies over time comparing anaerobic versus aerobic culture conditions. *T*. *scotoductus* cultures were grown anaerobically in a basal medium with lactate as the carbon source and nitrate as the terminal electron acceptor (top panel) or aerobically in TYG medium (bottom panel). Transcript and gene copies per mL of sample were calculated via absolute quantification using external calibration curves as described in the text. Error bars represent the standard deviation of triplicate qPCRs from three biological replicates.
(TIF)

S12 Fig *polA2* transcript and gene copies over time comparing oven versus microwave heating. *T*. *scotoductus* cultures were grown aerobically in TYG using oven (top panel) or microwave (bottom panel) heating. Transcript and gene copies per mL of sample were calculated via absolute quantification using external calibration curves as described in the text. Error bars represent the standard deviation of triplicate qPCRs from three biological replicates.
(TIF)

S13 Fig *polA2* transcript and gene copies after 24 h comparing terminal electron acceptors. *T*. *scotoductus* was grown anaerobically in basal medium with glucose as the carbon source and iron (Fe) or nitrate (NO_3_) as the terminal electron acceptor. Transcript and gene copies per mL of sample were calculated via absolute quantification using external calibration curves as described in the text. Error bars represent the standard deviation of triplicate qPCRs from three biological replicates.
(TIF)

S1 Table Standard curve details for *gyrB*, *polA2*, and *rpoC* assays, including primers used in standard construction, slope, y-intercept, r^2^, and PCR efficiency. (XLSX)

S2 Table MIQE checklist. (XLS)

S3 Table NormFinder intra- and intergroup variances under aerobic and anaerobic growth conditions. (XLSX)

S4 Table NormFinder intra- and intergroup variances during anaerobic growth with different terminal electron acceptors. (XLSX)

S5 Table NormFinder intra- and intergroup variances during aerobic growth with oven or microwave heating. (XLSX)

S6 Table NormFinder intra- and intergroup variances under all growth conditions. (XLSX)
