# Supplementary figures and images for: Selection and Evaluation of Reference Genes for Reverse Transcription-Quantitative PCR Expression Studies in a Thermophilic Bacterium Grown under Different Culture Conditions
Source: PLoS One. 2015 Jun 26;10(6):e0131015. doi: 10.1371/journal.pone.0131015 (PMC4482720; doi:10.1371/journal.pone.0131015)

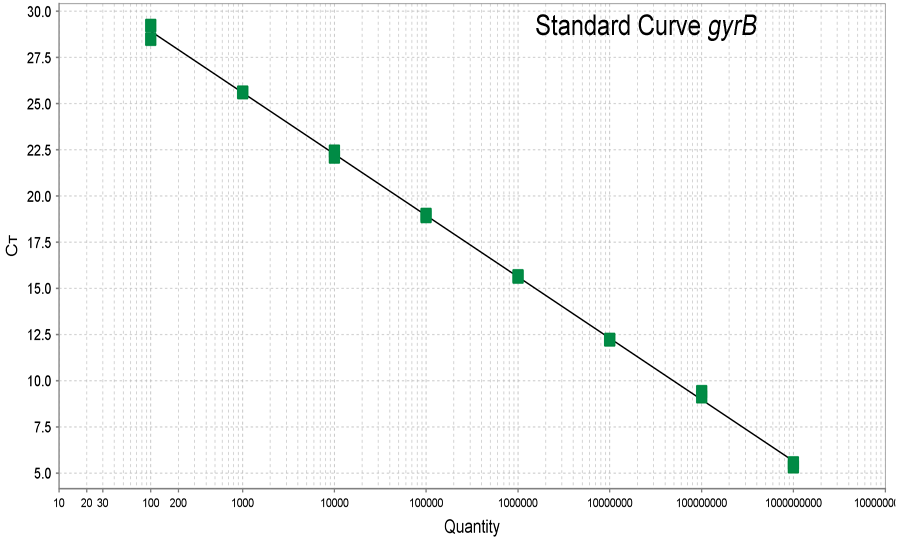

Supplement: S1 Fig — A 700 base pair region of the gyrase subunit B gene from the Thermus scotoductus genome was amplified using gene-specific primers. The resulting PCR product was purified and used in the construction of an external calibration curve. The number of copies per μL was calculated using the equation: X g μL-1 DNA/(PCR amplicon length x 660) x 6.022 x 1023. Serial 10-fold dilutions were prepared that spanned eight orders of magnitude ranging from 1 x 10^2^ to 1 x 10^9^ copies. The standard curve was determined by plotting the log of the calculated copy number against the cycle at which fluorescence for that sample crossed the threshold cycle. The slope, y-intercept, r2 value, and PCR efficiency of the gyrB assay is provided in S1 Table. (TIF) [file pone.0131015.s005.tif]

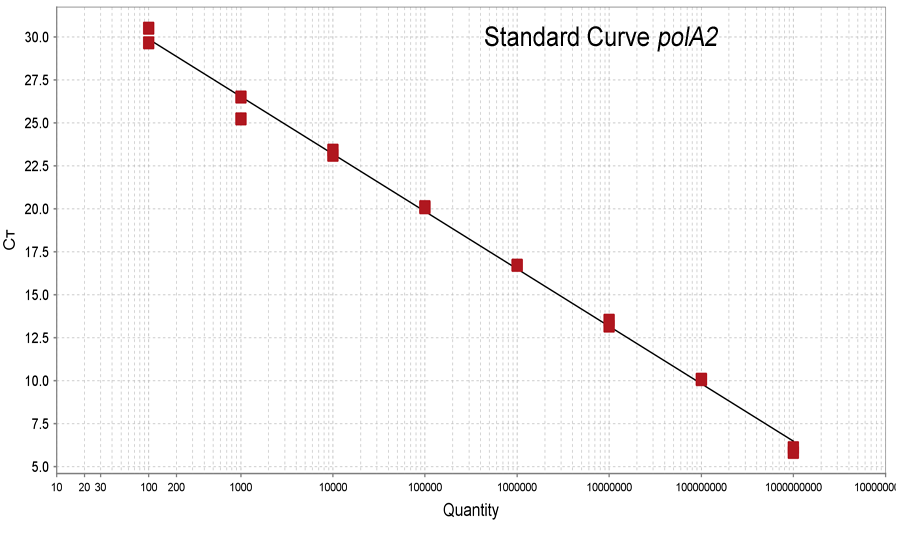

Supplement: S2 Fig — (TIF) [file pone.0131015.s006.tif]

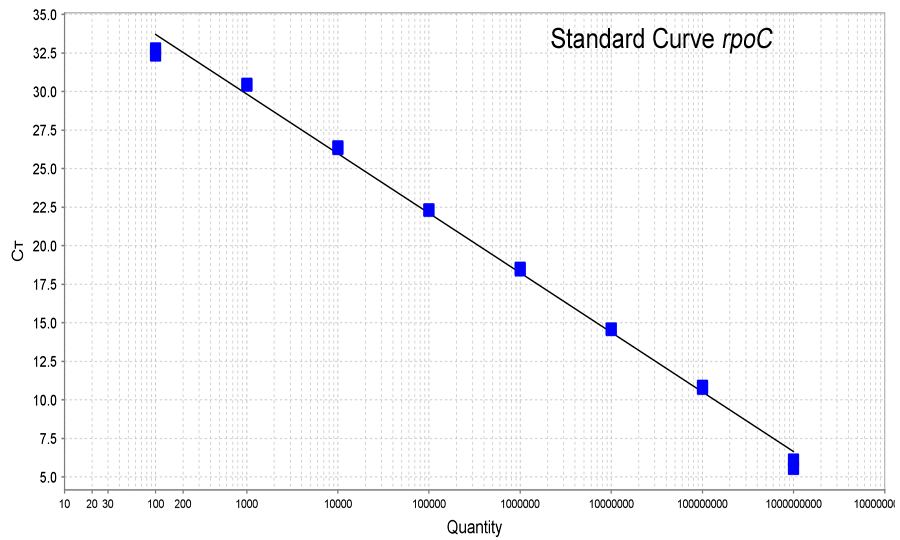

Supplement: S3 Fig — (TIF) [file pone.0131015.s007.tif]

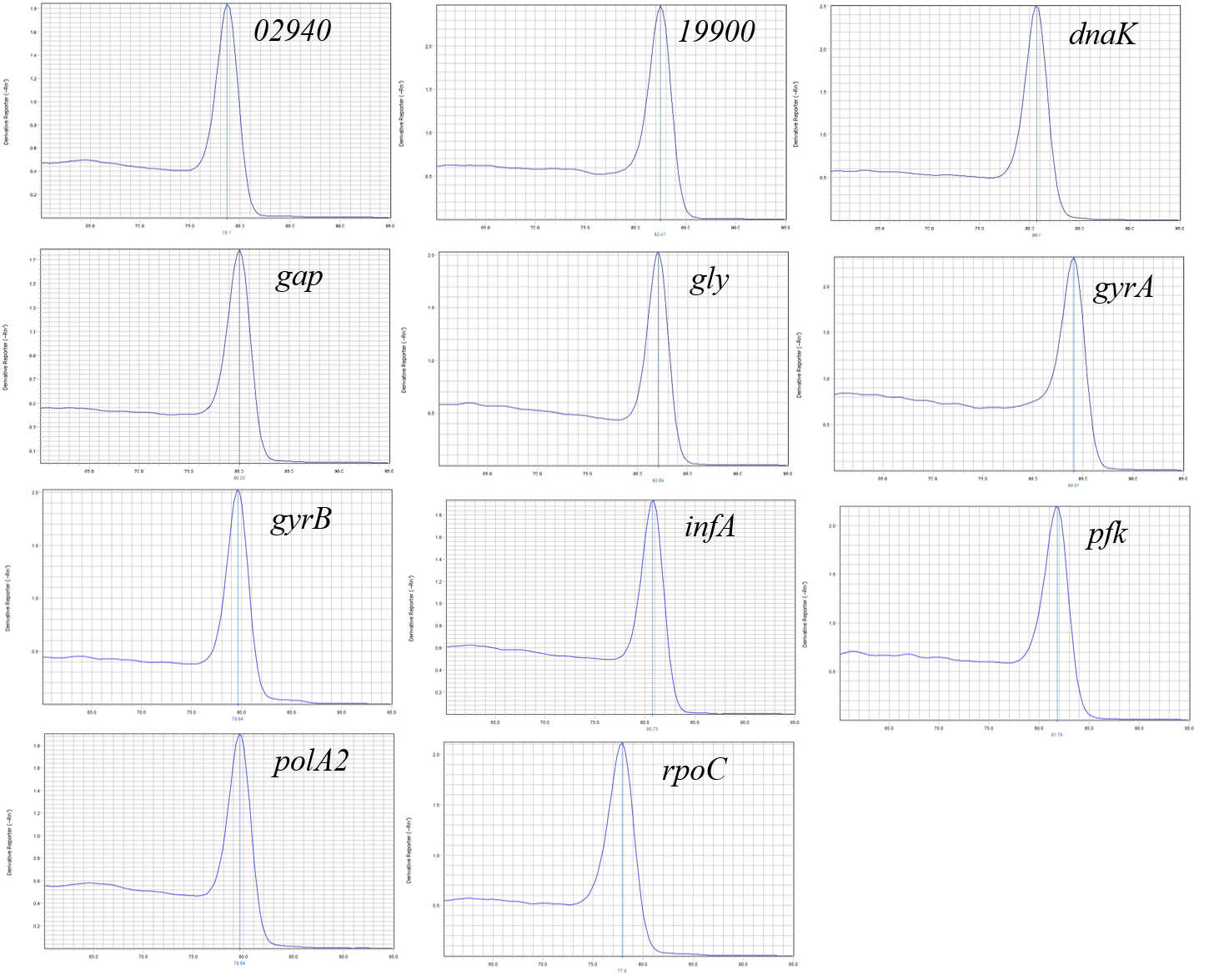

Supplement: S4 Fig — (TIF) [file pone.0131015.s008.tif]

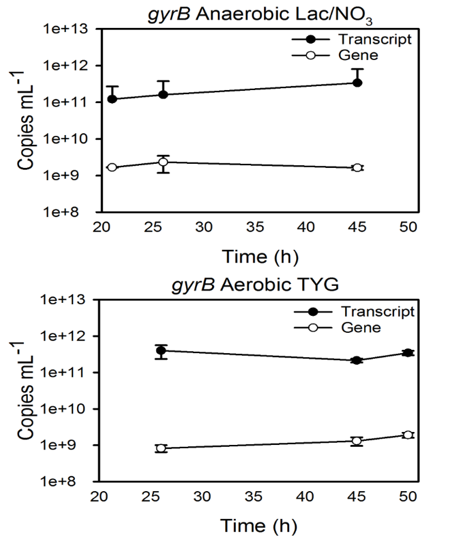

Supplement: S5 Fig — T. scotoductus cultures were grown anaerobically in a basal medium with lactate as the carbon source and nitrate as the terminal electron acceptor (top panel) or aerobically in TYG medium (bottom panel). Transcript and gene copies per mL of sample were calculated via absolute quantification using external calibration curves as described in the text. Error bars represent the standard deviation of triplicate qPCRs from three biological replicates. (TIF) [file pone.0131015.s009.tif]

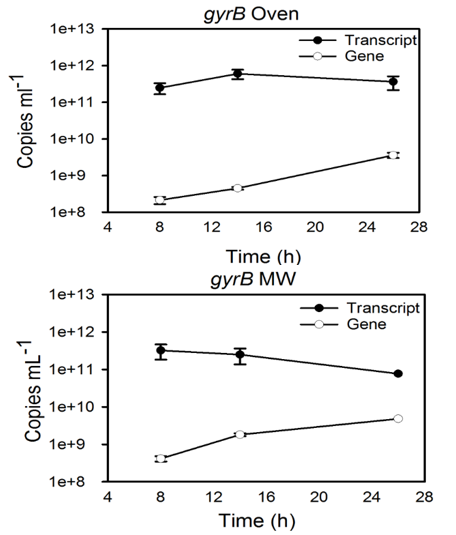

Supplement: S6 Fig — T. scotoductus cultures were grown aerobically in TYG using oven (top panel) or microwave (bottom panel) heating. Transcript and gene copies per mL of sample were calculated via absolute quantification using external calibration curves as described in the text. Error bars represent the standard deviation of triplicate qPCRs from three biological replicates. (TIF) [file pone.0131015.s010.tif]

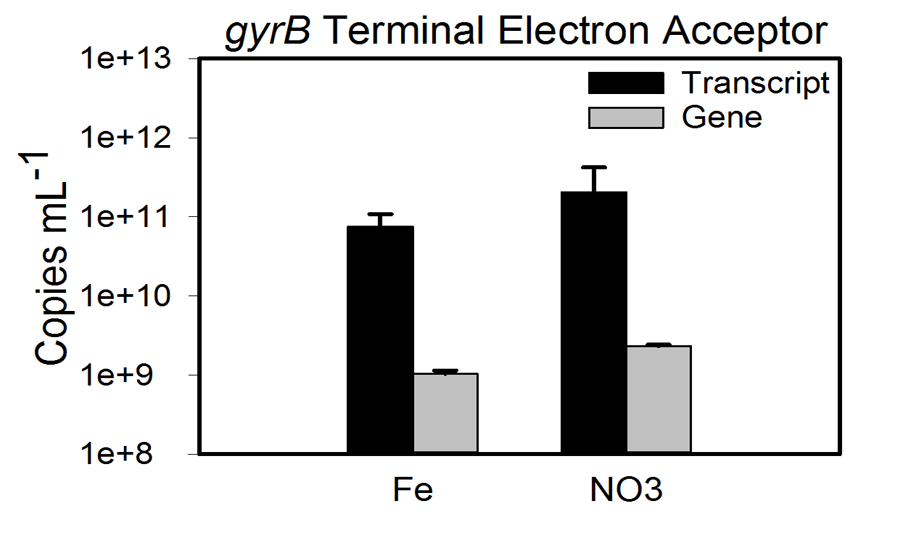

Supplement: S7 Fig — T. scotoductus was grown anaerobically in basal medium with glucose as the carbon source and iron (Fe) or nitrate (NO3) as the terminal electron acceptor. Transcript and gene copies per mL of sample were calculated via absolute quantification using external calibration curves as described in the text. Error bars represent the standard deviation of triplicate qPCRs from three biological replicates. (TIF) [file pone.0131015.s011.tif]

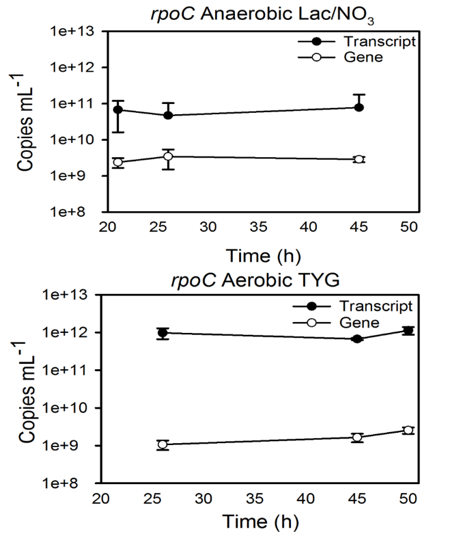

Supplement: S8 Fig — T. scotoductus cultures were grown anaerobically in a basal medium with lactate as the carbon source and nitrate as the terminal electron acceptor (top panel) or aerobically in TYG medium (bottom panel). Transcript and gene copies per mL of sample were calculated via absolute quantification using external calibration curves as described in the text. Error bars represent the standard deviation of triplicate qPCRs from three biological replicates. (TIF) [file pone.0131015.s012.tif]

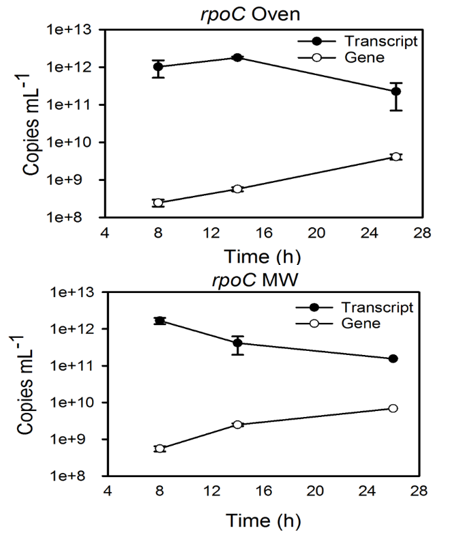

Supplement: S9 Fig — T. scotoductus cultures were grown aerobically in TYG using oven (top panel) or microwave (bottom panel) heating. Transcript and gene copies per mL of sample were calculated via absolute quantification using external calibration curves as described in the text. Error bars represent the standard deviation of triplicate qPCRs from three biological replicates. (TIF) [file pone.0131015.s013.tif]

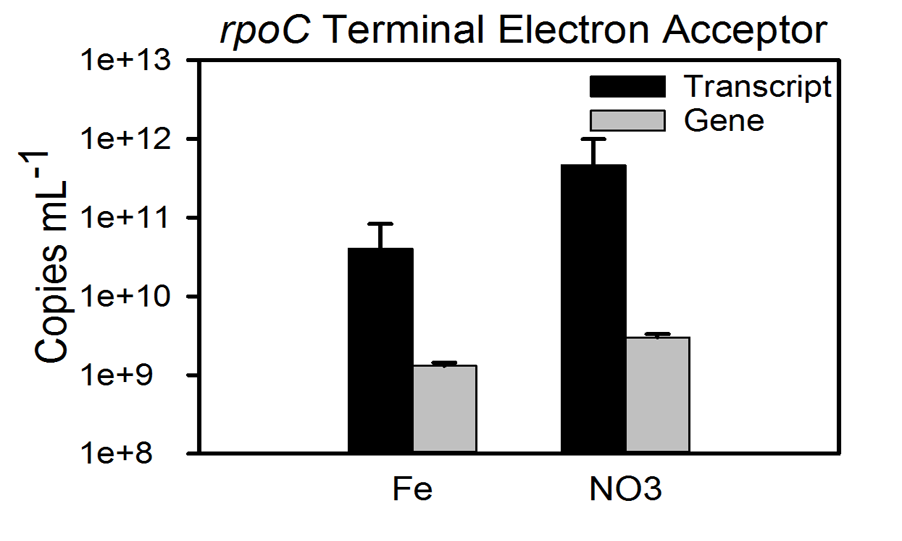

Supplement: S10 Fig — T. scotoductus was grown anaerobically in basal medium with glucose as the carbon source and iron (Fe) or nitrate (NO3) as the terminal electron acceptor. Transcript and gene copies per mL of sample were calculated via absolute quantification using external calibration curves as described in the text. Error bars represent the standard deviation of triplicate qPCRs from three biological replicates. (TIF) [file pone.0131015.s014.tif]

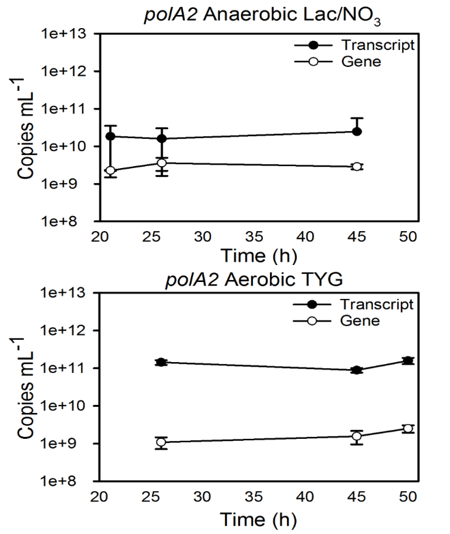

Supplement: S11 Fig — T. scotoductus cultures were grown anaerobically in a basal medium with lactate as the carbon source and nitrate as the terminal electron acceptor (top panel) or aerobically in TYG medium (bottom panel). Transcript and gene copies per mL of sample were calculated via absolute quantification using external calibration curves as described in the text. Error bars represent the standard deviation of triplicate qPCRs from three biological replicates. (TIF) [file pone.0131015.s015.tif]

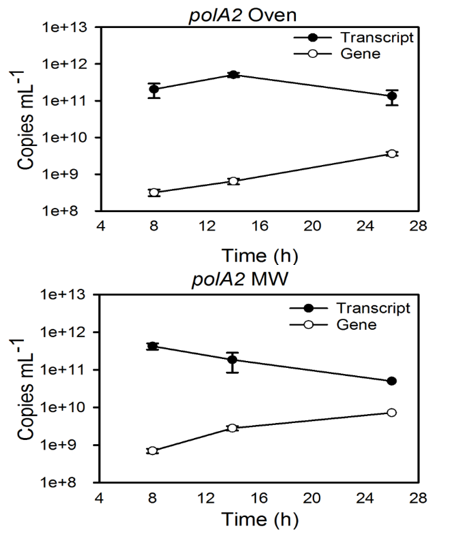

Supplement: S12 Fig — T. scotoductus cultures were grown aerobically in TYG using oven (top panel) or microwave (bottom panel) heating. Transcript and gene copies per mL of sample were calculated via absolute quantification using external calibration curves as described in the text. Error bars represent the standard deviation of triplicate qPCRs from three biological replicates. (TIF) [file pone.0131015.s016.tif]

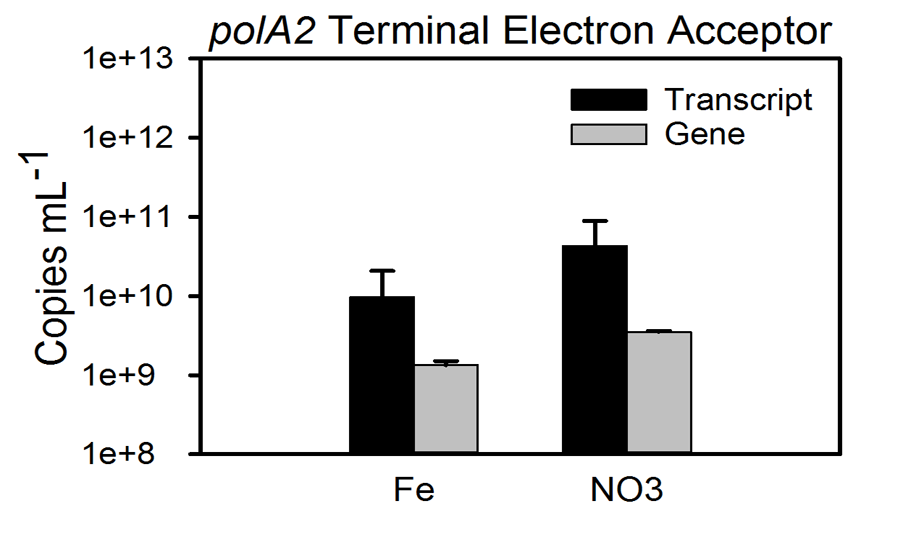

Supplement: S13 Fig — T. scotoductus was grown anaerobically in basal medium with glucose as the carbon source and iron (Fe) or nitrate (NO3) as the terminal electron acceptor. Transcript and gene copies per mL of sample were calculated via absolute quantification using external calibration curves as described in the text. Error bars represent the standard deviation of triplicate qPCRs from three biological replicates. (TIF) [file pone.0131015.s017.tif]
